# A case of pseudoxanthoma elasticum with proliferative diabetic retinopathy

**DOI:** 10.1186/s12886-017-0569-1

**Published:** 2017-10-04

**Authors:** Keigo Kakurai, Maiko Hayashi, Kanako Yamada, Norihiko Ishizaki, Yumiko Yonemoto, Seita Morishita, Ryohsuke Kohmoto, Takaki Sato, Teruyo Kida, Tsunehiko Ikeda

**Affiliations:** 1 Department of Ophthalmology, Osaka Medical College, 2-7 Daigaku-machi, Takatsuki City, Osaka, 569-8686 Japan; 2 Department of Ophthalmology, Yao Tokushukai General Hospital, Yao-City, Osaka, Japan; 3 Department of Ophthalmology, Hokusetsu General Hospital, Takatsuki-City, Osaka, Japan

**Keywords:** Pseudoxanthoma elasticum, Angioid streaks, Proliferative diabetic retinopathy, Vitrectomy

## Abstract

**Background:**

To report the case of a patient with pseudoxanthoma elasticum (PXE) and proliferative diabetic retinopathy (PDR), and discuss the relationship between PXE and diabetic retinopathy (DR).

**Case presentation:**

A 47-year-old man with PXE presented with angioid streaks and DR in both eyes, and bilateral panretinal photocoagulation was performed for treatment. Vitrectomy had previously been performed in his right eye for vitreous hemorrhage due to PDR. Systemic findings included multiple, discrete, symmetrical, small yellow papules bilaterally in the axilla and inguinal region. Examination on presentation showed vitreous hemorrhage in his left eye, and vitrectomy was performed for treatment. Intraoperative findings showed fibrovascular membrane around the optic disc and vascular arcade. A mottled fundus (peau d’orange appearance) associated with angioid streaks was also present, yet there was no evident choroidal neovascularization (CNV). The postoperative course was satisfactory, and corrected visual acuity improved from 0.02 to 0.7 diopters.

**Conclusion:**

Despite the peau d’orange appearance in both eyes of this case, no CNV was evident. The vitreous hemorrhage was thus attributed to PDR. Moreover, we reviewed the published literature and discuss the relationship between PXE and DR.

## Background

Pseudoxanthoma elasticum (PXE) is a genetic disorder characterized by calcification of elastic fibers, skin lesions, fundus lesions, and systemic vascular complications including coronary artery lesions, lower-leg arterial lesions, and gastrointestinal bleeding [1, 2]. The mode of inheritance in most families is autosomal recessive, but PXE with autosomal dominant inheritance has also been reported [3]. PXE affects approximately 1 in 160,000 people, typically appearing as a formation of yellow papules containing abnormally calcified elastic fibers. Cutaneous lesions are seen in the neck, axilla, cubital fossa, inguinal region, and periumbilical area. Angioid streaks are typically seen in the fundus due to breaks in the Bruch’s membrane [4].

Vascular findings in PXE mainly involve changes in medium-sized and large vessels [5], but microangiopathy has also been reported [6]. In this present study, we report an interesting case of PXE with diabetic retinopathy (DR), which is a microvascular disease. To the best of our knowledge, this is the first report of a case of proliferative diabetic retinopathy (PDR) complicated with PXE that required vitreous surgery.

## Case presentation

### Patient

A 47-year-old male.

### Chief complaint

Decreased vision in the left eye.

### History of present illness

This patient was being followed-up by a local physician for angioid streaks associated with PXE and bilateral DR. The DR had previously been treated with bilateral panretinal photocoagulation. In June 2002, pars plana vitrectomy was performed in his right eye by the previous physician for vitreous hemorrhage due to PDR. In January 2004, vitreous hemorrhage also occurred in the patient’s left eye. This temporarily regressed during follow-up, yet bleeding recurred in May 2004. The patient was subsequently referred to the Department of Ophthalmology at Osaka Medical College to undergo vitrectomy surgery.

### Past medical history

The patient had been diagnosed with type 2 diabetes in 2001. He began taking oral hypoglycemic drugs, and his hemoglobin (Hb)A1c was about 6% during follow-up. He was also taking antihypertensive drugs for treatment of hypertension. The patient had undergone craniotomy for a subdural hematoma in 1999, yet the details were unknown.

### Family history

None of the patient’s second-degree relatives had a history PXE. His father had diabetes, and his mother had hypertension.

### Findings on initial ocular examination

**Fig. 1:**
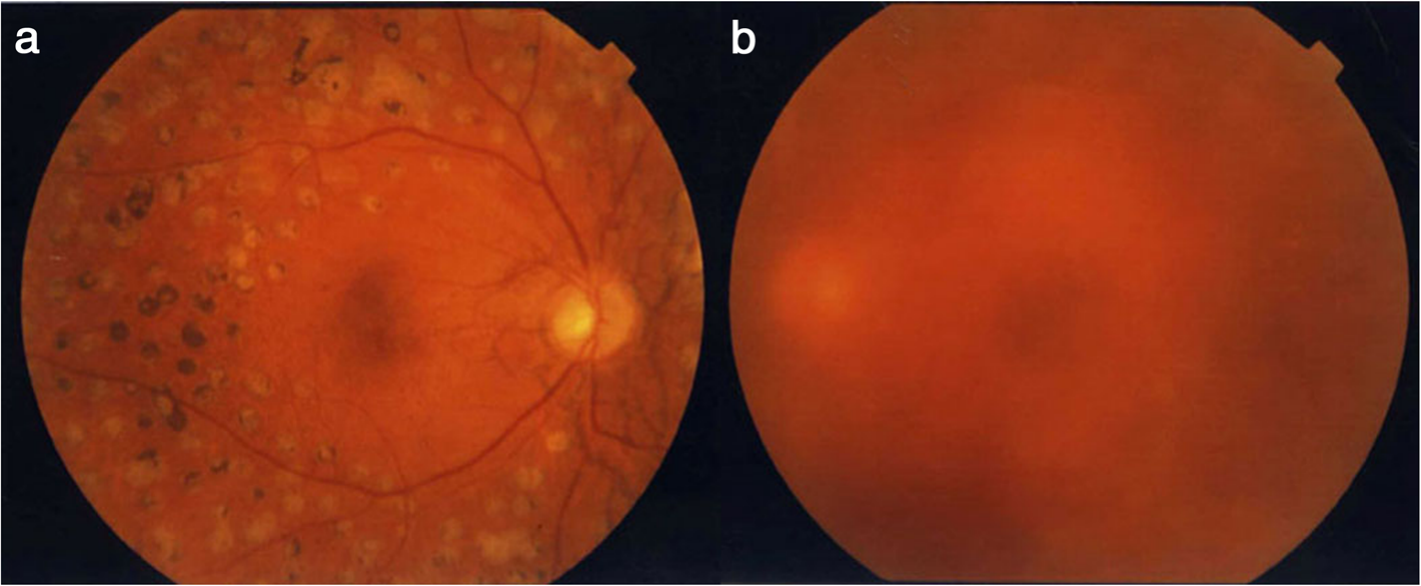
Preoperative fundus photographs (**a**: right eye; **b**: left eye). In the right eye, angioid streaks are evident around the optic disc, but no CNV is apparent. In the left eye, the fundus cannot be visualized due to vitreous hemorrhage

Upon initial ocular examination, the patient’s visual acuity (VA) was 0.04 diopters (D) (1.0 × S-11D = C-0.75D Ax15°)OD and 0.01D (0.02 × S-10.0D)OS, and his intraocular pressure (IOP) was 14 mmHg in both eyes. The anterior eye segments showed no abnormalities. Examination of the optic media showed mild nuclear cataracts bilaterally (Emery-Little classification: grade I nuclear sclerosis). Funduscopy of the right eye showed gray-white lines with rupture of the Bruch’s membrane extending radially from the optic disc (Fig. 1a). Some streaks extended to near the fovea, but no choroidal neovascularization (CNV) or subretinal hemorrhages involving the fovea were observed. The fundus displayed a peau-d’orange mottled appearance associated with angioid streaks. Vitreous hemorrhage in the patient’s left eye obscured the optic disc and the fundus could not be examined in detail (Fig. 1b).

### Findings on general physical examination

Multiple, discrete, symmetrical, small yellow papules were present bilaterally in the axillae and inguinal region. Rice-grain-sized yellowish-white papules were also present in a reticular pattern bilaterally on the neck. Blood-test results were as follows: WBC 9100/μl; hemoglobin (Hb) 10.8 g/dl; Htc 32.3%; Plt 342,000/μl; total protein 6.6 g/dL; GOT 15 units; GPT10 units; LDH 196 units; BUN 37 mg/dL; Cr 2.3 mg/dL; Na 143 mEq/L; K 5.2 mEq/L; Cl 107 mEq; HbA1c 6.3%; total cholesterol 213 mg/dL; and triglycerides 224 mg/dL.

### Follow-up course

**Fig. 2:**
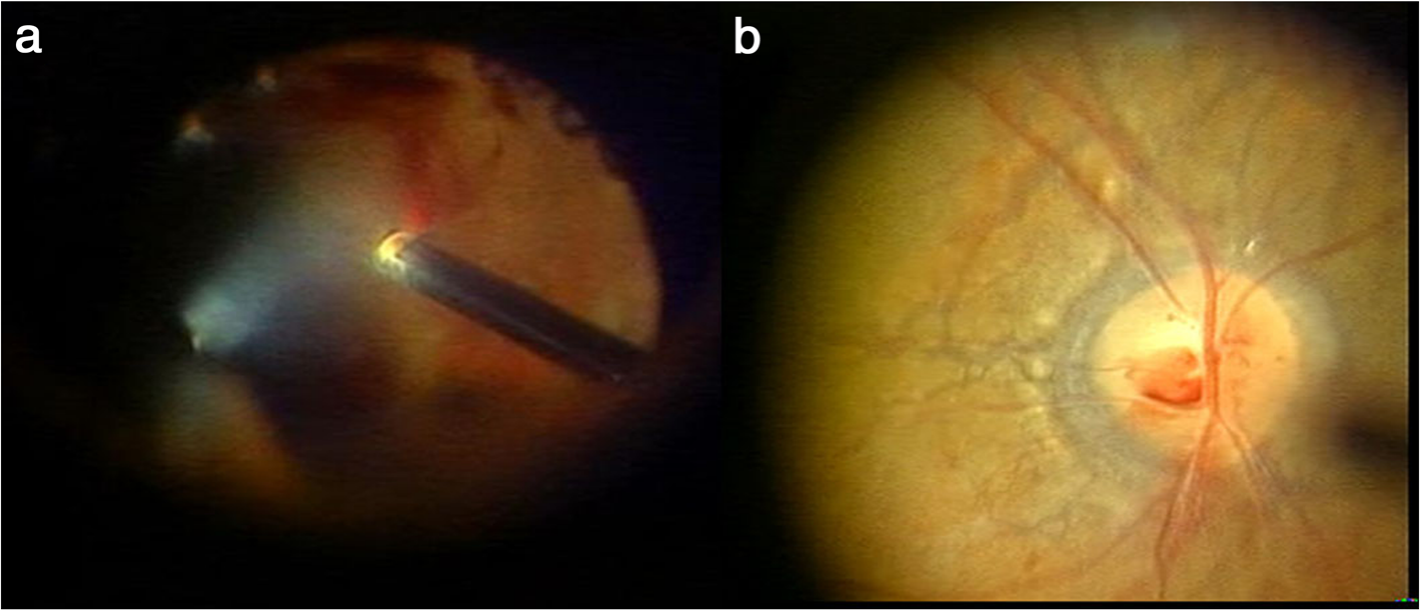
Intraoperative findings in the patient’s left eye. With removal of the vitreous hemorrhage (**a**), angioid streaks can be seen around the optic disc (**b**)

**Fig. 3:**
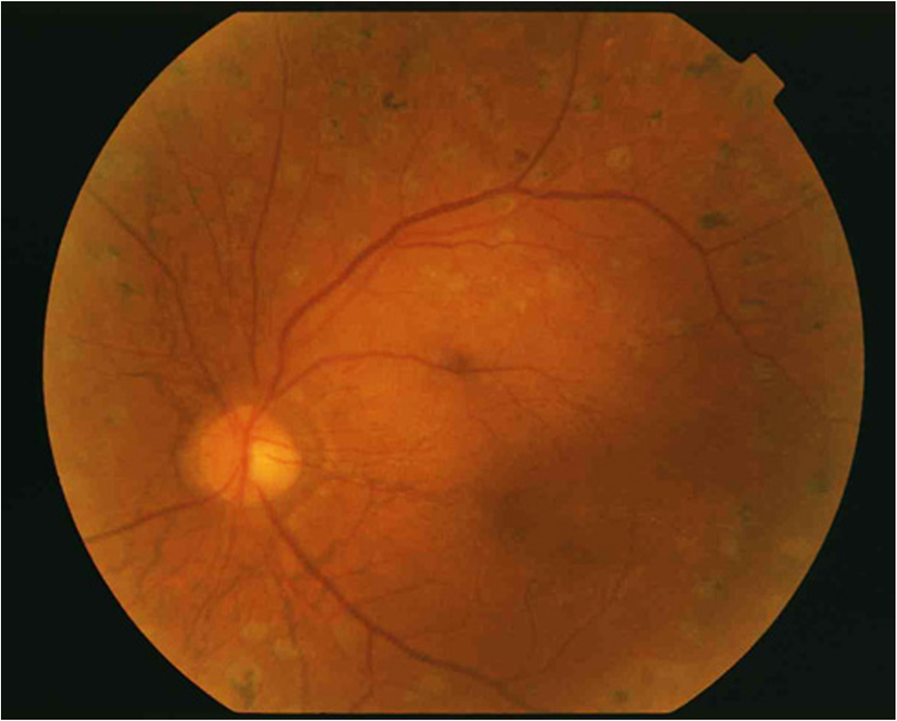
Postoperative fundus photograph of the patient’s left eye. The fundus is now visible, and corrected visual acuity has improved from 0.02 to 0.7 diopters

Pars plana vitrectomy for vitreous hemorrhage of the left eye was performed on June 16, 2004. Fibrovascular membrane was observed around the optic disc and superior and inferior vascular arcades; the vitreous humor was adherent at these sites, but posterior vitreous detachment (PVD) was evident in the surrounding area. The fibrovascular membrane was removed with a vitreous cutter and vitreous scissors, and the preretinal hemorrhage was aspirated (Fig. 2a). The fibrovascular membranes were nearly identical to those without PXE, and there were no unusual findings in this case. Peau d’orange appearance associated with angioid streaks around the optic disc, similar to that in the right fundus, was now observed (Fig. 2b). Breaks in the Bruch’s membrane extended to near the fovea, but no CNV was observed. Since the patient was young, the lens was preserved, the surrounding vitreous humor was removed, and intraocular photocoagulation was performed to complete the surgery. The postoperative course was good, visibility of the fundus improved, and corrected VA improved to 0.7D by 3-months after surgery(Fig. 3). At 36-months postoperative, the fundus remained stable with no occurrence of CNV.

## Discussion

PXE is a genetic disease characterized by a metabolic abnormality of elastin in elastic fibers, thus affecting organs with abundant elastic fibers such as the skin, eyes, and cardiovascular system [1, 2]. PXE with angioid streaks of the retina is also called Grönblad-Strandberg syndrome. The mode of inheritance may be either autosomal dominant or recessive, but autosomal recessive inheritance is the most common [3]. PXE is the most frequent systemic disorder associated with angioid streaks, occurring in about 1 in 100,000–200,000 people, and develops most often in patients between 20 and 30 years old. PXE progresses slowly, and the skin lesions include symmetrical small yellow papules in intertriginous areas such as the axilla, lateral neck, inguinal region, and on the abdomen. These papules gradually coalesce and aggregate, forming reticulated or cobblestone plaques. Due to the abnormal elastin fibers in various organs, complications often occur, including cardiovascular abnormalities, gastrointestinal bleeding, epistaxis, and intracerebral hemorrhage.

Histopathology shows calcium deposition in elastic fibers.

Angioid streaks are a degenerative disorder in which there is formation of radial streaks emanating from the optic disc due to degeneration and breaks in elastic fibers in the outer layer of the Bruch’s membrane. These occur in 80% of patients with PXE, most commonly in men. Angioid streaks usually occur in both eyes. Although the fundus is normal at birth, angioid streaks slowly progress and most often develop in patients between 40 and 50 years old. If the macula is not involved, few subjective symptoms are present and VA remains good. However, if the breaks in the Bruch’s membrane extend to the fovea, subretinal hemorrhage and fibrosis of the macula occur together with CNV, leading to metamorphopsia and central scotomas.

Patients with angioid streaks that do not extend to the macula can be followed-up, but treatment is necessary if CNV develops. If the CNV does not involve the macula, local laser photocoagulation can be performed, but the presence of subfoveal CNV requires anti-VEGF therapy and vitreous surgery to remove the neovascularization. Nevertheless, treatment outcomes generally remain poor. Systemic diseases other than PXE that are also associated with angioid streaks include Ehlers-Danlos syndrome, Paget’s disease of bone, and sickle cell disease [7].

In age-related macular degeneration and polypoidal choroidal vasculopathy, vitreous hemorrhage may occur due to CNV, but the cause of vitreous hemorrhage in our patient was probably PDR. Diabetes associated with PXE and angioid streaks has previously been reported [8, 9]. PXE usually causes systemic lesions involving medium-sized and large vessels, but systemic microangiopathy has also been reported [6], suggesting a causal relationship with DR.

Navarro-Lopez et al. [5] histopathologically examined cardiovascular tissue in PXE and reported arteriosclerosis and obstructive lesions due to degeneration and calcification of elastic fibers in the internal elastic lamina. This results in complications such as myocardial infarction, angina pectoris, arteriosclerosis obliterans, and gastrointestinal bleeding. Similar changes have also been reported in cerebral blood vessels [10]. In Mönckeberg’s arteriosclerosis with calcium deposition, the relative risk of cerebral infarction is increased [11]. The retina, like the brain, is part of the central nervous system, and similar mechanisms may also be involved in abnormalities of retinal blood vessels.

Recent research suggests that crosstalk between various factors can dynamically transform the cells constituting the vessel walls towards increased calcification, and diabetes is one factor that is known to promote calcification [12, 13]. Besides cell transformation, apoptosis also plays a role in calcification of mesenchymal cells in vessel walls (mainly vascular smooth muscle cells) [14]. Studies to date have reported these findings in relatively large vessels of the heart and kidneys, but similar mechanisms may be involved in small arterioles of the retinal vasculature. Pericyte loss is known to be important in the mechanisms of onset of DR. Pericyte apoptosis may also be deeply involved in calcification of retinal blood vessels.

An interesting report by Perdu et al., who used a capillary microscope system, described microangiopathy in a patient with PXE [6]. A capillary microscope is a specialized microscope used to observe capillaries and their blood flow in the fingertips. This type of system enables examination of capillaries without the need for painful procedures such as skin puncture and the drawing of blood [15]. Fingertip capillaries are very small, and the morphology of these vessels can undergo long-term changes due to the effects of lifestyle-related diseases such as diabetes. Perdu et al. used this technique to examine clinical and histological findings in 7 patients with PXE, and reported microangiopathy in all 7 patients.

## Conclusion

In conclusion, DR in patients with PXE has seldom been reported, but this may be because PXE itself is very rare. Taking into consideration the pathophysiology described above, systemic vascular changes in PXE may indeed play some role in the progression of DR.

## Abbreviations

CNV: Choroidal neovascularization
DR: Diabetic retinopathy
IOP: Intraocular pressure
PDR: Proliferative diabetic retinopathy
PXE: Pseudoxanthoma elasticum
